# Viral Interference of Hepatitis C and E Virus Replication in Novel Experimental Co-Infection Systems

**DOI:** 10.3390/cells11060927

**Published:** 2022-03-08

**Authors:** Thomas Burkard, Nora Proske, Kathrin Resner, Laura Collignon, Leonard Knegendorf, Martina Friesland, Lieven Verhoye, Ibrahim M. Sayed, Yannick Brüggemann, Maximilian K. Nocke, Patrick Behrendt, Heiner Wedemeyer, Philip Meuleman, Daniel Todt, Eike Steinmann

**Affiliations:** 1 Department of Molecular and Medical Virology, Ruhr University Bochum, 44801 Bochum, Germany; thomas.burkard@ruhr-uni-bochum.de (T.B.); yannick.brueggemann@ruhr-uni-bochum.de (Y.B.); maximilian.nocke@ruhr-uni-bochum.de (M.K.N.); 2 TWINCORE Centre for Experimental and Clinical Infection Research, a Joint Venture between the Medical School Hannover (MHH) and the Helmholtz Centre for Infection Research (HZI), Institute for Experimental Virology, 30625 Hannover, Germany; proske.nora@mh-hannover.de (N.P.); kathrin.resner@gmx.de (K.R.); knegendorf.leonard@mh-hannover.de (L.K.); martina.friesland@twincore.de (M.F.); patrick.behrendt@twincore.de (P.B.); 3 Department of Gastroenterology, Hepatology and Endocrinology, Hannover Medical School, 30625 Hannover, Germany; wedemeyer.heiner@mh-hannover.de; 4 Laboratory of Liver Infectious Diseases, Ghent University, 9000 Gent, Belgium; laura.collignon@ugent.be (L.C.); lieven.verhoye@ugent.be (L.V.); ibrahim.ibrahim@aun.edu.eg (I.M.S.); 5 Microbiology and Immunology Department, Faculty of Medicine, Assiut University, Assiut 71515, Egypt; 6 German Centre for Infection Research (DZIF), Partner Site Hannover Braunschweig, 30625 Hannover, Germany; 7 European Virus Bioinformatics Center (EVBC), 07743 Jena, Germany; 8 German Centre for Infection Research (DZIF), External Partner Site, 44801 Bochum, Germany

**Keywords:** Hepatitis C virus (HCV), Hepatitis E virus (HEV), co-infection, human hepatocytes, sofosbuvir, HCV protease, human liver chimeric mice

## Abstract

Background: Hepatitis C virus (HCV) constitutes a global health problem, while hepatitis E virus (HEV) is the major cause of acute viral hepatitis globally. HCV/HEV co-infections have been poorly characterized, as they are hampered by the lack of robust HEV cell culture systems. This study developed experimental models to study HCV/HEV co-infections and investigate viral interference in cells and humanized mice. Methods: We used state-of-the art human hepatocytes tissue culture models to assess HEV and HCV replication in co- or super-transfection settings. Findings were confirmed by co- and super-infection experiments in human hepatocytes and in vivo in human liver chimeric mice. Results: HEV was inhibited by concurrent HCV replication in human hepatocytes. This exclusion phenotype was linked to the protease activity of HCV. These findings were corroborated by the fact that in HEV on HCV super-infected mice, HEV viral loads were reduced in individual mice. Similarly, HCV on HEV super-infected mice showed reduced HCV viral loads. Conclusion: Direct interference of both viruses with HCV NS3/4A as the determinant was observed. In vivo, we detected reduced replication of both viruses after super-infection in individual mice. These findings provide new insights into the pathogenesis of HCV-HEV co-infections and should contribute to its clinical management in the future.

## 1. Introduction

Hepatitis E virus (HEV) is a member of the *Orthohepevirus* genus within the family of *Hepeviridae*. The positive-sense RNA virus is an understudied pathogen, which accounts for approximately 20 million annual infections [1] and thus constitutes a major global health burden [2]. Virus strains harmful to humans mainly belong to the species *Orthohepevirus-A*, genotypes 1–4. HEV-1 and 2 are transmitted via the fecal–oral route, are endemic in countries with poor sanitation standards and mainly lead to sporadic outbreaks and acute hepatitis. In contrast, HEV-3 and 4 are mostly zoonotic and spread by close contact to infected animals or consumption of their undercooked meat. While often self-limiting, infection can evolve into chronicity in immunocompromised patients, of which solid-organ transplant (SOT) recipients are the most studied population [3]. Besides tapering the immunosuppression, the therapy of chronic HEV is limited to off-label use of ribavirin and, in rare cases, peg-interferon [4]. It has been demonstrated that ribavirin therapy fails to achieve a sustained virological response (SVR) in about 20% of patients [5,6]. To date, there is no specific antiviral therapy against HEV available, which in part is due to the past inability to efficiently propagate HEV in tissue culture [7,8,9,10].

Hepatitis C virus (HCV) is a positive-sense RNA virus belonging to the *Hepacivirus* genus in the *Flaviviridae* family. HCV is distributed worldwide with geographic differences in genotype prevalence [11]. Despite the appearance of direct acting antivirals (DAA) with efficacies >90%, HCV continues to be a serious health threat, affecting an estimated 184 million patients worldwide [12]. Its primary transmission route is percutaneous exposure to blood, such as iatrogenic infections or the use of contaminated devices for drug injection [13]. Persistent HCV infection is associated with chronic inflammation [14], which over time leads to hepatic injuries ranging from minimal necro-inflammatory changes up to fibrosis and cirrhosis that may result in liver decompensation [15]. Major issues preventing its eradication are HCV being underdiagnosed and, for low-income countries, poor treatment affordability [12]. Even in high-income countries, efforts in eradication are not on track everywhere, with 80% of high-income countries being expected to fail HCV elimination targets for 2030 [16]. Therefore, HCV will likely remain a public health burden.

Given the population-based abundance of HEV and HCV, co-infections of these two viruses would be expected to occur frequently in selected patients. HCV and HEV are both underdiagnosed pathogens. The percentage of diagnosed HCV cases has been estimated at 36.4% for the EU [17] and 12.2% for the US [18], respectively. On the other hand, the number of reported HEV cases is low, despite studies suggesting high pervasiveness. A meta-analysis found HEV seroprevalence to range from 7.5% to 31.9% in different EU countries [19], while screening of blood donors suggests that 1 in 3000 people have an active HEV infection [20]. These data imply that there might be many overlooked infections and possibly co-infections with unknown influence of clinical course. However, only three case reports have described active HCV/HEV co-infections so far [21,22,23], highlighting that they are not well documented. Two patients had recurrent HCV infections after liver transplantation with HEV infection of unknown origin. One received sofosbuvir, daclatasvir and ribavirin and cleared both viruses [21], while the other patient was treated with sofosbuvir and daclatasvir for 12 weeks and achieved an SVR for HCV, but not for HEV [22]. The third patient had a triple infection of HCV, HEV and hepatitis B Virus (HBV) and achieved an SVR by a 12-week regimen of tenofovir, daclatasvir, sofosbuvir and ribavirin [23]. The aim of this study was to gain a deeper insight into the phenomenon of HCV/HEV co-infection. So far, viral interferences or interactions between these two hepatitis viruses have not been investigated due to the lack of appropriate experimental model systems. This limitation has recently been overcome by the establishment of an efficient and robust HEV cell culture system [8], allowing for the first time to characterize HCV and HEV co-infections employing a variety of experimental model systems.

## 2. Materials and Methods

### 2.1. Plasmids

pFK_i389Lucubineo_NS3-3′_dg_JFH-1 [24], pFKi389Neo-NS3 3′_dg_JFH 1 _NS5Aaa2359_RFP [25], pFK-Jc1 [26], pWPI_FLAG-JFH-1-NS34A and pWPI_FLAG-JFH-1-NS34A-S139A [27] were previously described. The subgenomic Kernow-C1 p6 ***Gaussia*** luciferase (GLuc) [28] and GFP replicon [8], the subgenomic Sar55 S17 GLuc replicon [29], the subgenomic HEV83-2-27 GLuc replicon [30] (kind gift of the laboratory of Takaji Wakita) were all previously described. The Kernow-C1 p6 GFP-Neo plasmid was cloned from a Kernow C1 p6 GLuc-Neo template (kindly provided by Viet Loan Dao Thi) by replacing the GLuc gene with a GFP.

### 2.2. Compounds and Reagents

Ribavirin was received from Sigma Aldrich (St. Louis, MO, USA). Sofosbuvir and telaprevir were purchased from MedChemExpress (Monmouth Junction, NJ, USA). 2 C′ methyladenosine (2′-CMA) was a gift from Timothy Tellinghuisen (The Scripps, Jupiter, FL, USA). All compounds were diluted in DMSO and stored at −80 °C.

### 2.3. Cell Culture

The human liver cell lines Huh7.5 and Huh7-Lunet were cultured in Dulbecco’s modified Eagle’s medium (DMEM) (Invitrogen, Karlsruhe, Germany) supplemented with 10% fetal calf serum (FCS, GE Healthcare, Chicago, IL, USA), 100 μg/mL of streptomycin and 100 IU/mL of penicillin (Invitrogen), 2 mM L-glutamine and 1% nonessential amino acids (Invitrogen) (DMEM complete). Huh7-Lunet cells stably expressing pFKi389Neo-NS3 3′_dg_JFH 1_NS5Aaa2359_RFP (Huh7-Lunet sg/neo) have been previously described [25]. Huh7.5-p6-GFP-Neo cells were produced by transfecting IVTs from Kernow-C1 p6 GFP Neo into Huh7.5 cells. Huh7.5 cells overexpressing NS3/4A constructs and the RFP-NLS-IPS sensor were produced by transduction of Huh7.5_RFP NLS-IPS cells with lentiviral pseudoparticles. All cells were selected and maintained in DMEM supplemented with 750 µg/mL G418 Sulfate (ThermoFisher Scientific, Waltham, MA, USA). Cells were kept at 37 °C and 5% CO_2_.

### 2.4. In Vitro Transcription and Electroporation

All HCV containing plasmids as well as HEV Kernow-C1 p6 were linearized using MluI (New England Biolabs, Fankfurt a. M., Germany), HEV 83-2-containing plasmids were linearized with HindIII (New England Biolabs), and Sar55-containing plasmid with EcoRV (New England Biolabs). HCV-based plasmids were in vitro transcribed as indicated [31]. HEV-based plasmids were transcribed as indicated [8]. For transfection we used the electroporation technique in accordance to the previous reports [28]. In brief, 5 × 10^6^ Huh7.5 cells in 400 µL cytomix containing 2 mM ATP and 5 mM glutathione were mixed with a total of 5 µg of RNA. Electroporation was carried out with a Gene Pulser system (Bio-Rad, Munich, Germany). Cells were immediately transferred to 12.1 mL of DMEM complete and the cell suspension was seeded in respective plates depending on the experiment (2 × 10^4^ cells/well seeded in a 96-well plate for luciferase assays, 2 × 10^5^ cells/well in 12-well plates for flow cytometry analysis and 7 × 10^4^ cells/well in 24 well plates for immunofluorescence (IF) analysis).

### 2.5. Luciferase Assay

Compounds were added 4 h post electroporation (h p.e.). For measuring GLuc, 20 µL of supernatant were collected at indicated timepoints and transferred to a white, flat-bottom microplate (Greiner Bio-One, Solingen, Germany Ref. 655074). For measuring *Firefly* luciferase (FLuc), cells were washed once with PBS and taken up in 20 µL lysis buffer (containing 0.1% Triton-X100, 25 mmol/L glycylglycine, 15 mmol/L MgSO4, 4 mmol/L EGTA tetrasodium, and 1 mmol/L dithiothreitol, pH 7.8) and lysed via freeze-thaw and then transferred to a white, flat-bottom microplate. Supernatants were incubated with luciferase substrate (1 μmol/L of coelenterazin in PBS, P.J.K Biotech, Kleinblittersdorf, Germany) and luciferase activity was measured in a luminometer (CentroXS3 LB960, Berthold technologies, Bad Wildbad, Germany). Cell lysates were incubated with luciferase substrate (200 μmol/L luciferin, 25 mmol/L glycylglycine, pH 8) and measured with a luminometer (CentroXS3 LB960, Berthold technologies).

### 2.6. Immunofluorescence Staining

Cells were fixed at the end of respective incubation time in 3% PFA solution for at least 10 min at room temperature before permeabilization with 0.2% Triton X-100 in PBS and blocking by 5% horse serum (ThermoFisher Scientific, 26050-088) in PBS at room temperature for at least 1 h. For co-infection and sequential infection experiments, HEV capsid was stained with rabbit anti-HEV-ORF2 serum (kind gift of Prof. Rainer G. Ulrich, Friedrich Loeffler Institute, Germany, 1/4000 in PBS supplemented with 5% horse serum), while for super-infection experiments, HEV was stained with rabbit anti-GFP (Invitrogen, A11122, 1/1000 in PBS supplemented with 5% horse serum). HCV was stained with murine anti-HCV-NS5A-9E10 antibody (kind gift of Prof. Charles M. Rice, Rockefeller University, New York, USA, 1/10,000 in PBS supplemented with 5% horse serum), all overnight at 4 °C. HEV secondary staining was performed with Alexa 488-labeled goat anti-rabbit IgG (Invitrogen A11008, 1/1000 in PBS supplemented with 5% horse serum), HCV with Alexa 555-labeled donkey anti-mouse IgG (Invitrogen, A32773, 1/1000 in PBS supplemented with 5% horse serum). Plates were imaged at an Olympus IX81 microscope (Olympus) (transfection experiments) or a Keyence BZ X810 (Keyence Deutschland GmbH, Neu-Isenburg, Germany).

### 2.7. Quantification of HCV and HEV-Positive Cells in Fluorescence Microscopy Pictures

Mean fluorescence intensities of HCV immunofluorescence were obtained from a 5-pixel wide cytoplasm ring (cytoring) following segmentation of DAPI-stained nuclei using CellProfiler. HEV immunofluorescence intensities were also obtained from this cytoring. To distinguish noninfected cells from infected cells, minimum intensity thresholds were applied. Cells were classified into four categories: positive for both HCV and HEV, positive for HCV only, positive for HEV only or double negative.2.8. Western Blot.

Cells were detached with trypsin and taken up in DMEM, washed once with PBS and taken up in SDS sample buffer. After freeze-thaw, samples were digested with 1 µL benzonase (25–29 Units/µL, Millipore, Burlington, MA, USA; 70664-10KUN) until solution was easy to pipet. Samples were boiled at 98 °C for 5 min. A total of 300,000 cells each were separated on an SDS PAGE and immobilized on a polyvinylidene fluoride membrane (GE Healthcare #10600023). Membrane was blocked in PBS with 0.5% Tween20 (PBS-T), supplemented with 5% skimmed milk powder. NS3 was detected with murine anti NS3 #337 F3A6 (homemade, 1/500) for 3.5 h at room temperature, ß actin with anti-murine anti ß-actin antibody (Sigma-Aldrich, St. Louis, MO, USA; A2228, 1/1000) for 1 h at room temperature. Bound primary antibodies were detected by incubation with anti-mouse-IgG-HRP (Sigma, A4416, 1/20,000) for 1 h at room temperature.

### 2.8. VSV Infection

The vesicular stomatitis virus was kindly provided by Dr. Gert Zimmer [32]. Cells were infected with VSV-GFP for 16 h followed by fixation with 3% paraformaldehyde (PFA).

### 2.9. Flow Cytometry

After respective incubation times, cells were trypsinized, washed and fixed in PBS supplemented with 1% FCS and 0.5% PFA for 10 min at room temperature. Cells were analyzed at a BD Acurri C6 Plus FACS (BD Biosciences, Franklin Lakes, NJ, USA).

### 2.10. Pseudoparticle Production for Production of Transduced Cell Lines

Lentiviral pseudoparticles were produced by transfecting pcz-VSV-G, pCMV-dR8.74 and pWPI with the respective gene of interest into HEK 293T cells with Lipofectamine 2000 (Thermo Fisher, 11668019). Supernatants were harvested after 24 and 48 h, pooled and filtered through 0.45 µM filters (Sarstedt, Nümbrecht, Germany; Filtropur 0.45, 83.1826) und used to infect target cells. Target cells were selected and maintained in DMEM complete, containing Geneticin (G418-Sulfate, ThermoFisher Scientific, 11811031, 750 µg/mL final concentration).

### 2.11. HCVcc and HEVcc Production

For infectious cell-culture derived HCV (HCVcc) production, HCV IVTs were electroporated into Huh7.5 cells. Supernatant was harvested 48 and 72 h post electroporation, pooled and centrifuged at 200× *g*. Virus titer was determined by TCID50 and aliquoted and frozen at −80 °C until usage. For infectious cell culture derived HEV (HEVcc) production, HEV IVTs were electroporated into HepG2 cells. Cells were trypsinized and resuspended in fresh DMEM 7 days post electroporation and lysed via three freeze thaw cycles. The lysate was cleared from cell debris by a 10,000× *g* centrifugation for 10 min and titrated on HepG2/C3A cells to determine viral titers. Virus was frozen at −80 °C until usage.

### 2.12. HEV Super-Transfection Experiments

To prepare Huh7-Lunet cells stably expressing NS3 3′_dg_ JFH-1NS5Aaa2359_RFP for super-transfection experiments, G418 selection was discontinued with simultaneous introduction of sofosbuvir, ribavirin or DMSO treatment. Cells were cultivated under these conditions for 3 days before electroporation and subsequently cultivated for 5 days before subjecting cells to IF or flow cytometry analysis. For DAA treatment of HCV selected replicons, selection antibiotic was removed, and cells were treated with indicated drugs for 5 days before super-transfection with HEV.

### 2.13. HEV Infection Experiments

For all infection experiments, 2 × 10^4^ cells/well were seeded in 24-well plates and allowed to adhere overnight. Infection was performed the next day. Virus inoculum was removed after 24 h. Cells were either fixed 4 days post infection (d p.i.) or received a second virus infection for 24 h followed by 4 days incubation and subsequent fixation and preparation for IF. All infections were carried out with a multiplicity of infection of 1.

### 2.14. HCV Super-Infection on HEV Expressing Cells

A total of 2 × 10^4^ cells/well were seeded in 24-well plates and allowed to adhere overnight without G418 selection agent. Infection was performed the next day and inoculum was replaced after 24 h with DMEM. 5 d p.i., cells were fixed with PFA and prepared for IF.

### 2.15. Determination of Viral Titers

To determine titers of infectious HCV, tissue culture infectious dose (TCID_50_/mL) assay was performed, as described previously [33]. To determine infectious HEV titers, focus forming units were determined as described [8].

### 2.16. Production and Infection of Human Liver Chimeric Mice

uPA**^+/+^**-SCID mice were transplanted with cryopreserved primary human hepatocytes (PHH), as previously described [34]. Successful engraftment was assessed by human albumin concentration in mouse plasma, determined by ELISA (Bethyl Laboratories, Montgomery, TX, USA). Mice were either intraperitoneally injected with a fecal suspension containing 10^6^ IU HEV-3f or intrasplenically with a plasma sample containing 1.5 × 10^5^ IUHCV GT 1a. Fecal and blood samples were routinely collected and stored at −80 °C until analysis. The animal study protocol was approved by the Animal Ethics Committee of the Faculty of Medicine and Health Sciences of Ghent University. HEV and HCV RNA was extracted from fecal or plasma samples, respectively, using the NucliSENS easyMAG system (Biomérieux, Craponne, France). HEV RT qPCR was performed as described by Sayed et al. [35]. HCV RNA was detected using the RealStar^®^ HCV RT PCR kit (Altona, Hamburg, Germany) and the LightCycler 480 (Roche Diagnostics Deutschland GmbH, Mannheim, Germany).

### 2.17. Statistical Analysis and Graphics

Statistical analysis was performed using GraphPad Prism v9.12 for Windows (La Jolla, CA, USA, www.graphpad.com) *p* values < 0.05 (*), <0.01 (**), <0.001 (***) and <0.0001 (****) were considered statistically significant. *p* values >0.05 were considered to be non-significant (ns). Graphics were prepared using GraphPad Prism v9.12 for Windows (La Jolla, CA, USA, www.graphpad.com), Adobe Illustrator v26.0.3 (www.adobe.com) and BioRender (www.biorender.com, accessed on 3 February 2022).

## 3. Results

### 3.1. HCV Impairs HEV Replication in Subgenomic Reporter Co-Transfection Assays

**Figure 1:**
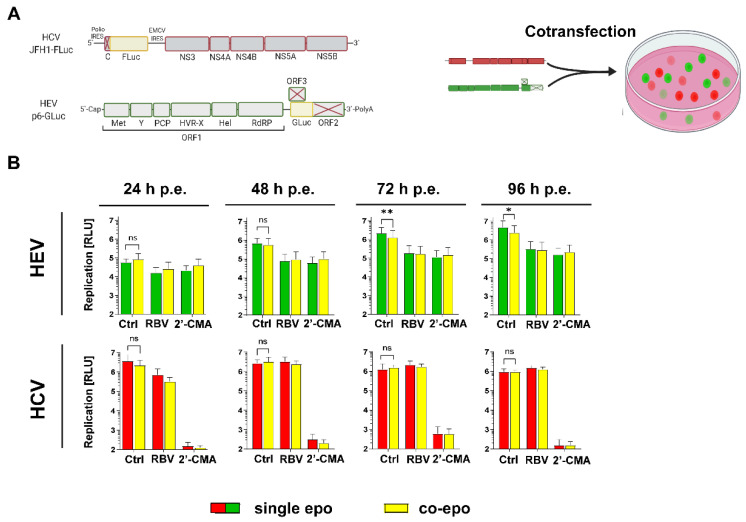
Co-replication of HCV and HEV in Huh7.5 cells. (**A**) Scheme of the subgenomic replicon constructs used for co-replication studies. (**B**) Huh7.5 cells were transfected with HEV p6 subgenomic replicon harboring a *Gaussia* luciferase and HCV subgenomc replicons harboring a *Firefly* luciferase reporter gene for dual luciferase measurement. Shown are respective luciferase counts in samples harvested at the indicated h post electroporation (h p.e.). A total of 10 µM 2′ C methyladenosine (2′ CMA, HCV) and 25 µM ribavirin (RBV, HEV) were added 4 h post electroporation, as replication controls for the respective viruses. Depicted are mean ± SD from three independent experiments. (Statistical significance was calculated using a one-tailed paired *t*-test). * = *p*-value < 0.05; ** = *p*-value < 0.01.

To test whether HEV and HCV influence each other’s replication when introduced into the same cell population, subgenomic replicons harboring either a *Gaussia* (HEV) or *Firefly* (HCV) luciferase reporter were transfected into Huh7.5 cells by electroporation, either alone or together (Figure 1A). *Gaussia* luciferase is secreted into the supernatant, while *Firefly* luciferase remains intracellular, enabling dual-luciferase measurements to simultaneously monitor HCV and HEV replication kinetics. Ribavirin (HEV) and 2′-C-methyladenosine (HCV) were included as inhibitors for the respective viruses. Of note, 2′-C-methyladenosine exhibited also anti-HEV properties, which is in line with findings from the literature [36]. HEV replication was significantly reduced after 72 and 96 h post transfection when compared to single transfection (Figure 1B, upper panels), while HCV replication was not affected (Figure 1B, lower panels). These results indicate that HEV replication was influenced by co-replicating HCV, but not vice versa.

**Figure 2:**
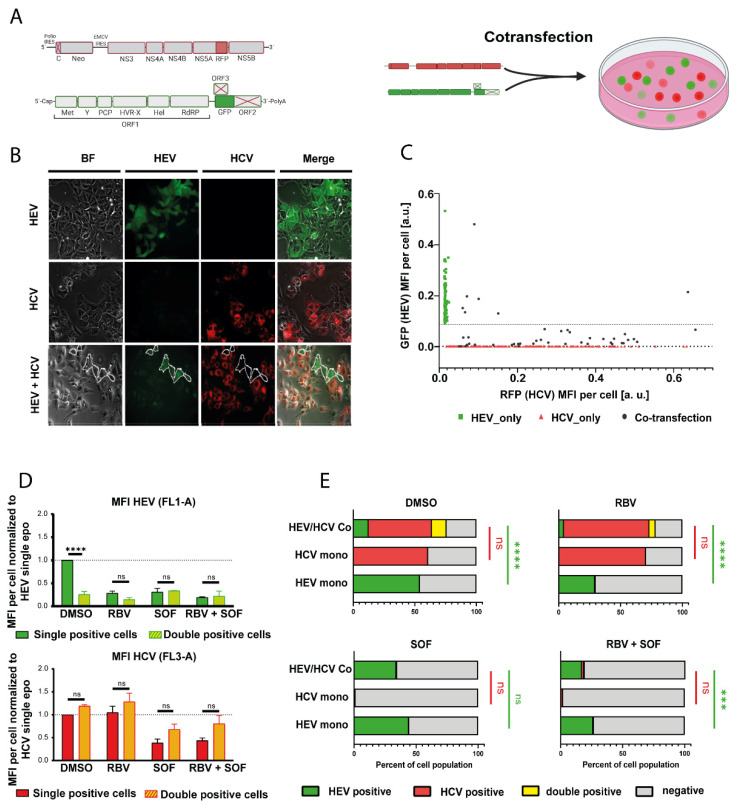
Co-transfection of fluorescently labeled HCV and HEV reporter replicons. (**A**) Scheme of the subgenomic replicon constructs and the experimental design used for experiments. (**B**) Representative IF pictures of Huh7.5 hepatoma cells transfected with the fluorescence reporters HEV p6 GFP and HCV subgenomic replicon JFH1-NS5A-RFP. HEV-positive cells in the HCV/HEV condition are encircled. Cells were imaged 5 days post electroporation (**C**) Plot of GFP mean fluorescence intensity (MFI) vs. RFP MFI of individual cells from 2B. Each point stands for a single cell. (**D**) Co transfected cells were analyzed by flow cytometry 5 days post electroporation. Single and double positive cells were compared for their mean fluorescence intensities of HEV (upper panel) or HCV (lower panel) signal. Cells were incubated with either DMSO or 25 µM ribavirin (RBV), 10 µM sofosbuvir (SOF) or 25 µM ribavirin and 10 µM sofosbuvir (RBV + SOF). Treatment was started 4 h post electroporation. Depicted are mean ± SD. (Two-way ANOVA with Dunnet’s multiple comparison test). (**E**) Co-transfected cells were analyzed by flow cytometry 5 days post electroporation for the percentage of cells positive for HEV, HCV or both, as determined by flow cytometry. Cells were incubated with either DMSO or 25 µM RBV, 10 µM SOF or 25 µM RBV and 10 µM SOF. Treatment was started 4 h post electroporation. Depicted are mean ± SD. (Two-way ANOVA with Sidak’s multiple comparison test). *** = *p* value < 0.001; **** = *p* value < 0.0001.

Measurements of virus-encoded luciferases only provide information on the scale of a cell population and thus do not allow us to distinguish whether the inhibition of HEV replication was due to a lower fraction of HEV-transfected cells and/or lower replication levels. To address this question, we co-transfected a GFP expressing HEV replicon based on Kernow-C1 p6 strain (p6-GFP) and an RFP expressing HCV replicon based on JFH-1 (JFH-1-RFP) into Huh7.5 cells (Figure 2A). Microscopy analysis showed only a few HEV positive cells in the HCV/HEV co-transfected setting, as compared to cells only transfected with HEV (Figure 2B). Conversely, the number of HCV positive cells was not affected (Figure 2B). Comparison of the respective GFP and RFP fluorescence intensities on a single cell level further revealed strongly reduced levels for GFP upon co-transfection with HCV, as compared to cells only transfected with HEV (Figure 2C), indicative of lowered HEV replication. Flow cytometry analysis was used to quantify the inhibitory effect. HEV/HCV double positive cells had approximately 70% reduced GFP levels while the RFP signal was not changed (Figure 2D and Figure S1). Similarly, the number of GFP positive cells dropped from 54% to 12%, while the number of RFP positive cells was unaffected (Figure 2E, upper left panel). The effect of HCV on HEV was dependent on active HCV replication, as concomitant sofosbuvir treatment reverted the percentage of GFP positive cells back to levels similar to single HEV transfection (Figure 2E, lower left panel). This was also the case for the co-treatment of sofosbuvir with ribavirin (Figure 2E, lower right panel). In summary, these results demonstrate that HEV replication is hindered in HCV-replicating cells, while HCV replication is not influenced by the presence of HEV subgenomic replicons.

### 3.2. HCV Treatment Restores HEV Replication Capacity

**Figure 3:**
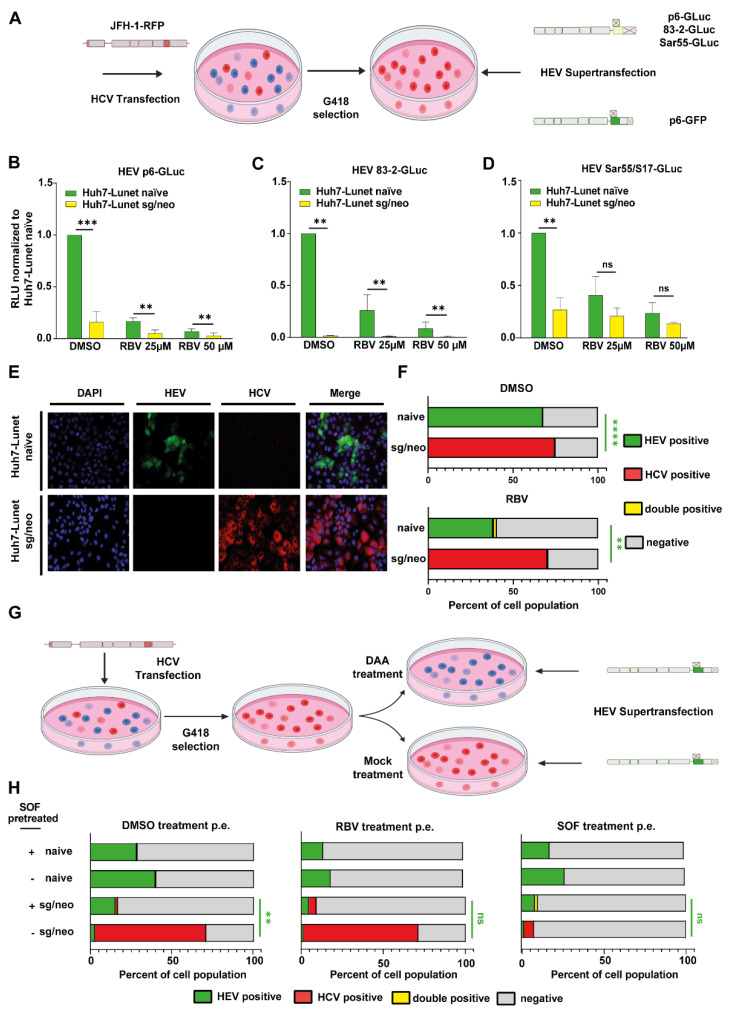
HEV replication in HCV-selected human hepatocytes. (**A**) Schematic representation of experimental setup for panels (**B**–**F**). Huh7 cells were transfected with a selectable HCV subgenomic replicon expressing RFP, JFH1-NS5A-RFP, and selected and subsequently transfected with either an HEV luciferase reporter or GFP reporter subgenomic replicon. G418 treatment was discontinued upon transfection to rule out unspecific effects on HEV. (**B**–**D**) Replication of HEV replicons 72 h post electroporation was assessed in naïve Huh7-Lunet cells or Huh7-Lunet cells expressing the selectable HCV subgenomic replicon (Huh7-Lunet/sg-neo). Cells were incubated with DMSO or ribavirin (RBV). Treatment was started 4 h post electroporation. Depicted are means ± SD from three independent experiments. (Two-way ANOVA with Sidak’s multiple comparison test). (**E**) Representative IF pictures of Huh7-Lunet naïve (upper panel) or Huh7-Lunet/sg-neo cells (lower panel) transfected with a HEV p6-GFP subgenomic reporter replicon, imaged 5 days post electroporation. (**F**) Percentage of Huh7-Lunet naïve or Huh7-Lunet/sg-neo cells positive for HCV, HEV, both or none, as determined by flow cytometry 5 days post electroporation. Cells were treated with DMSO or 25 µM RBV. Depicted are means from three independent replicates. (Two-way ANOVA with Sidak’s multiple comparison test of positive cell percentages). (**G**) Schematic representation of experimental setup for panel (**H**). Cells were transfected with JFH1-NS5A-RFP, selected as in (**B**–**F**) and subsequently either cured with 10 µM sofosbuvir (DAA treatment) or treated with 0.1% DMSO (mock treatment) for 72 h prior to transfection of HEV subgenomic replicons. (**H**) Percentage of Huh7 Lunet naïve or Huh7-Lunet/sg-neo cells positive for HCV, HEV, both or none was assessed by flow cytometry 5 days post electroporation of HEV. Cells were treated with DMSO, 25 µM RBV or 10 µM sofosbuvir (SOF). Treatment was started 4 h post electroporation. Depicted are means ± SD of three independent experiments. (Two-way ANOVA with Sidak’s multiple comparison test of positive cell percentages). ** = *p* value < 0.01, *** = *p* value < 0.001; **** = *p* value < 0.0001.

In a clinical context, it is likely that an active infection with both viruses does not occur simultaneously, but rather consecutively. Therefore, Huh7 Lunet cells were transfected with a subgenomic JFH-1 replicon harboring an NS5A-RFP and a neomycin resistance cassette (Huh7 Lunet sg/neo), allowing super-transfection with luciferase-encoding HEV of fluorescently labeled HCV-positive cells (Figure 3A). The selected cells were used to test HCV’s capacity to interfere with different HEV isolates, including Kernow C1 p6, 83-2-27 (HEV-3) and Sar-55 (HEV-1). Replication was reduced for all HEV strains between 70% (Sar55) and 98% (83-2-27) in Huh7 Lunet sg/neo cells compared to Huh7-Lunet naïve cells, indicating that the previously observed effects are not co-transfected as well as not HEV-3 exclusive (Figure 3B–D). Additionally, p6 GFP was super-transfected into Huh7-Lunet naïve and Huh7-Lunet-sg/neo cells. While establishing replication in 68% of naïve Huh7-Lunets, HEV did only replicate in 0.5% of cells that had been selected for HCV (Figure 3E,F and Figure S3), confirming the inhibitory effect of HCV on HEV-replication. Super-infection of Huh7 Lunet naïve or Huh7 Lunet sg/neo with vesicular stomatitis virus (VSV) did not result in either reduced HCV or VSV signal (Figure S2), indicating a specific viral interference with HEV. To test if clearance of HCV would render hepatocytes susceptible to HEV again, HCV positive cells were either treated with sofosbuvir or DMSO for 5 days and subsequently super-transfected with HEV and analyzed via flow cytometry (Figure 3G and Figure S4). DAA pretreatment significantly enhanced the number of HEV positive cells from 2.5% to 15%, while simultaneously reducing HCV positive cells from 70% to 1.4% (Figure 3H; left panel). To exclude a potential antiviral effect of sofosbuvir on HEV [37,38], these results were confirmed with telaprevir (Figure S5). These data suggest that the exclusion phenotype of HEV replication required active HCV replication and could be reverted by clearance of HCV with DAAs.

### 3.3. The HCV Protease NS3/4A Inhibits HEV Replication

**Figure 4:**
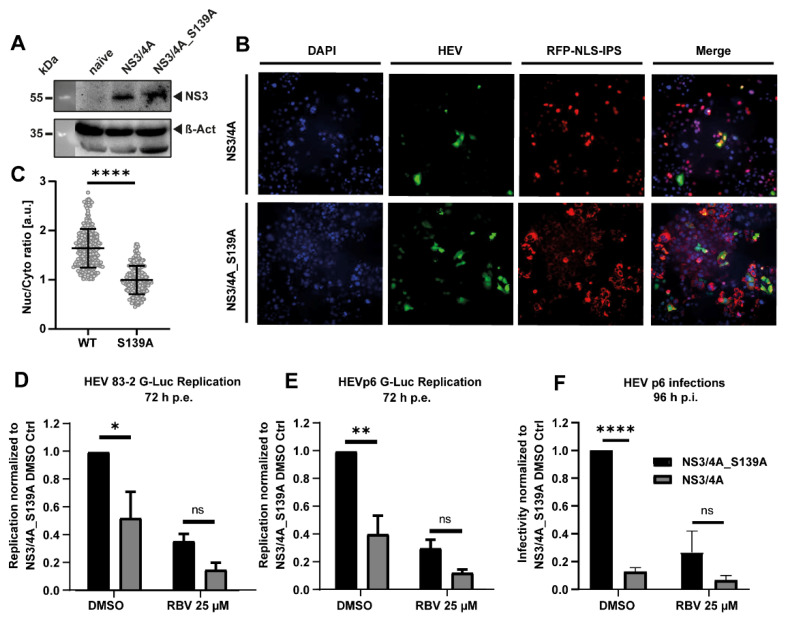
HEV replication after ectopic HCV protease NS3/4A expression. (**A**) Western blot of Huh7.5 cells overexpressing NS3/4A or NS3/4A_S139A. (**B**) Representative IF pictures of Huh7.5_RFP-NLS-IPS-NS3/4A cells 3 days post electroporation. (**C**) Single cells were analyzed for the subcellular localization of RFP. The ratio of analysis of nuclear (Nuc) divided by cytosolic (Cyto) signal was calculated based on IF pictures in (**B**). More than 200 cells were analyzed for each condition. Depicted are mean ratios of cells from one experiment. (Two-tailed unpaired *t*-test). (**D**,**E**) Replication levels of different HEV *Gaussia* luciferase subgenomic reporter replicons in Huh7.5 NS3/4A and NS3/4A_S139A 72 h post electroporation. (Two-way ANOVA with Sidak’s multiple comparison test on normalized relative luminescence units (RLU)). Cells were incubated with DMSO or ribavirin (RBV). Treatment was started 4 h post electroporation. Depicted are means ± SD from three independent experiments. (**F**) Relative susceptibility of Huh7.5 NS3/4A and NS3/4A_S139A cells to HEVcc. Cells were treated with DMSO or RBV. 96 h post infection and cells were analyzed with immunofluorescence against HEV capsid protein ORF2. The number of foci was manually counted. Depicted are means ± SD from three independent experiments. Treatment was started together with infection start. (Two-way ANOVA with Sidak’s multiple comparison test on normalized infection events). * = *p* value < 0.05, ** = *p* value < 0.01; **** = *p* value < 0.0001.

To identify a potential determinant for the HCV-mediated restriction of HEV replication, we next generated Huh7.5 cells constitutively expressing the HCV protease NS3/4A as a wildtype protein or as catalytically inactive S139A mutant. The expression of the protein was determined by Western blot (Figure 4A). The functionality of the proteins was validated by expression of a sensor consisting of a fusion protein of RFP and the NS3/4A substrate interferon-β promotor stimulator 1 (IPS-1), which are linked via a nuclear localization sequence (NLS) [39]. If a functional NS3/4A protein is expressed, IPS-1 is cleaved, exposing the NLS and resulting in the translocation of RFP into the nucleus. Representative IF pictures and the quantitative determination of the nuclear-localized RFP signal verified the successful expression of operative wildytpe NS3/4A in Huh7.5 cells, in contrast to Huh7.5 cells expressing the S139A mutant (Figure 4B,C). Next, we analyzed the interference of the HCV protease with HEV replication. NS3/4A alone was sufficient to inhibit the replication of subgenomic luciferase replicons of both 83-2-27 (Figure 4D) as well as Kernow-C1 p6 (Figure 4E). This inhibitory effect was even more pronounced when infecting HCV protease expressing cells with infectious cell-culture derived HEV (HEVcc) (Figure 4F). These data indicate that the HCV protease NS3/4A was sufficient to inhibit HEV replication and infection.

### 3.4. HCV Super-Infection of HEV-Replicating Cells

**Figure 5:**
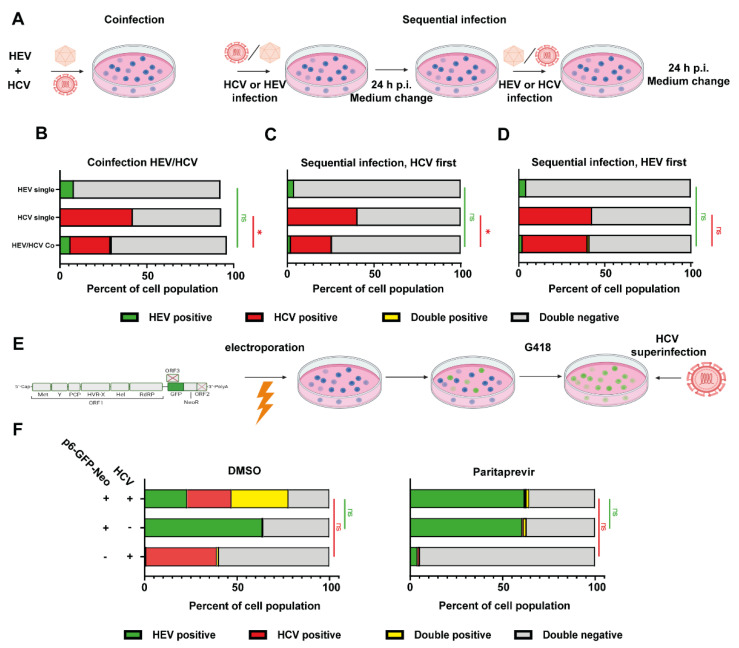
Co-infection and sequential super-infection with HCVcc and HEVcc. (**A**) Schematic representation of experimental setup for panels (**B**–**D**): Huh7.5 cells were co-infected with HEVcc (based on Kernow-C1 p6 strain) and HCVcc (based on Jc1). HEV and HCV inoculum were incubated on the cells for 24 h, either together (**B**), or sequentially (**C**,**D**). Four days post infection, cells were analyzed with immunofluorescence against HEV capsid protein ORF2. The number of foci was manually counted. Depicted are mean percentage ± SD of positive cells from three independent experiments for HEV, HCV, none or both as determined via image analysis with Cell Profiler. (Two-way ANOVA with Tukey’s multiple comparison test on normalized infection events). (**E**) Schematic representation of experimental setup for panel (**F**): Huh7.5 hepatoma cells were transfected with a HEV subgenomic GFP-reporter replicon comprised of all non-structural proteins of HEV Kernow-C1 p6. Cells were selected and maintained analogous to Huh7 Lunet sg/neo cells. (**F**) Five days after super-infection with HCV, the cells were analyzed by immunofluorescence. Graphs show mean percentage of positive cells ± SD of three independent experiments for HEV, HCV, none or both as determined via image analysis with Cell Profiler. Cells were treated with DMSO or 10 µM paritaprevir. Treatment was started 4 h post electroporation. (Two-way ANOVA with Tukey’s multiple comparison test on normalized infection events). * = *p* value < 0.05.

As we recently established improved cell culture conditions to generate HEVcc, we next investigated viral interference in an authentic HCV/HEV co-infection setup. A co-infection approach inoculating Huh7.5 cells with HCV and HEV at the same time as well as a sequential infection was conducted as depicted (Figure 5A). In the co-infection condition, the percentage of HEV-positive cells was not significantly altered, whereas the number of HCV-infected cells was reduced from around 40% in single infections to 20% (Figure 5B). In the sequential infection approaches, HEV infectivity was not changed, infecting approximately 5% of cells (Figure 5C,D). However, HCV-positive cells were reduced when HCV infected first from around 40% to 25%, but remained unaffected when HEV infected first (Figure 5C,D). As these experimental model systems might not fully reflect the potentially long timeframes between consecutive viral infections, we generated Huh7.5 cells that harbor a neomycin-selectable GFP-labeled p6 replicon to mimic a chronically HEV-infected stage before HCV super-infection (Figure 5E). IF analysis demonstrated that approximately 65% of Huh7.5 cells selected (Huh7.5 p6-GFP-Neo) were positive for HEV (Figure 5F). The percentage of HCV positive cells was around 40% both in naïve Huh7.5 cells as well in Huh7.5 p6 GFP-Neo cells, implying that HCV can super-infect HEV replicating cells and its infectivity was unaffected by HEV (Figure 5F). Treatment with the NS3/4A targeting DAA paritaprevir reduced the number of HCV positive cells and had no influence on HEV replication (Figure 5F). These results show that HCV can co-infect human hepatocytes with HEV and super-infect cells harboring a selectable HEV replicon.

### 3.5. HEV or HCV-Positive Human Liver Chimeric Mice Showed Reduced Viral Loads in Individual Mice after Super-Infection

**Figure 6:**
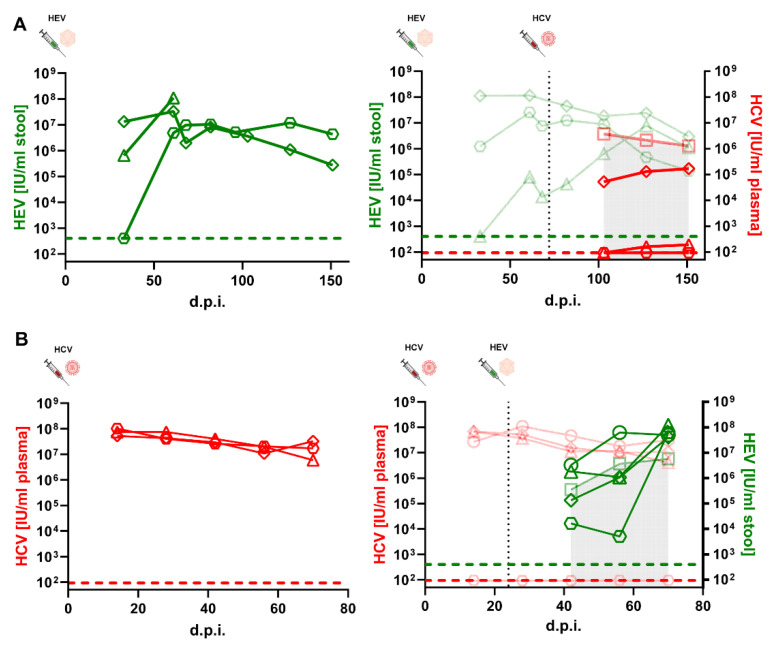
HCV or HEV super-infections on HEV- or HCV-infected humanized mice. (**A**) Human liver chimeric uPA^+/+^-SCID mice were injected intraperitoneally with HEV and subsequently injected intravenously with HCV (black dashed line). HEV RNA (green data points) and HCV RNA (red data points) were periodically measured. Left panel: HEV viral loads of HEV mono-infected mice. Right panel: HEV viral loads of HEV co-infected animals as well as mean HCV titers of HCV mono-infected (semitransparent data points) and HCV titers of HCV co-infected mice. Green dashed line and red dashed line indicate the limit of detection (LOD) of the HEV or HCV RT qPCR, respectively. (**B**) Human liver chimeric uPA^+/+^-SCID mice were injected intravenously with HCV and subsequently injected intraperitoneally with HEV (black dashed line). HEV RNA (green data points) and HCV RNA (red data points) were periodically measured. Left panel: HCV viral loads of HCV mono-infected mice. Right panel: HCV titers of HCV co-infected animals as well as mean HEV viral loads of HEV mono-infected (semitransparent data points) and HEV viral loads of HEV co infected mice. Green dashed line and red dashed line indicate the LOD of the HEV or HCV RT qPCR, respectively.

Human liver chimeric mice have been established as an animal model to study both HEV [35,40] as well as HCV (reviewed in [41]). We used homozygous urokinase plasminogen activator (uPA)-SCID^+/+^ mice, of which the liver was reconstituted by primary human hepatocytes (PHH) to analyze HCV/HEV co-infections in vivo. To this end, animals were either inoculated intraperitoneally with HEV or intravenously with HCV. After several weeks, which allowed for the establishment of the infection, the animals were super-infected with the respective other virus. Viral loads were determined by RT qPCR from stool (HEV) or plasma (HCV) at regular intervals. Mice only infected with HEV (Figure 6A, left panel) showed viral loads between 10^6^ and 10^8^ IU/mL after the onset of infection and stabilized over the time monitored. Viral loads developed similarly in HEV positive mice super-infected with HCV (Figure 6A, right panel). In contrast, HCV could not establish productive infection in two out of three HEV infected mice, while in the other mouse reduced viral loads were observed compared to the HCV mono-infection group visualized by the semitransparent red line underlined with a grey area (Figure 6A, right panel). Restriction of HCV infection in HEV-positive mice was also observed when the HCV super-infection was conducted at an earlier time point (Figure S6).

For the HEV on HCV super-infection protocol, mice only infected with HCV had viral loads between 10^7^ and 10^8^ IU/mL at all time points (Figure 6B, left panel). HCV viral loads developed similarly in HCV positive mice super-infected with HEV, except for one mouse that could not be infected at all (Figure 6B, right panel). HEV viral loads were reduced and delayed in two individual mice, while in the two other HCV super-infected mice comparable or higher HEV copy numbers could be observed (Figure 6B, right panel). In sum, viral interference in the replication kinetics of HCV and HEV was observed in individual mice after super-infection.

## 4. Discussion

Although HCV and HEV are both important pathogens with a considerable disease burden that targets the same organ, clinical information on HCV/HEV co-infections is limited, while molecular characterizations are absent. To address this gap in knowledge, we investigated HCV/HEV co-infections in vitro and in vivo. To address co-infection in vitro, a transfection model and an infection model were used that differed in several aspects and thus have distinct advantages. The transfection model’s advantage is that it guarantees high percentages of HEV- and HCV-positive cells. This high percentage is important, because it statistically increases the number of cells, where both viruses aim to establish an infection and is hence more sensitive observing viral interference. The co-infection model with an infectious virus is more complex, as it does reflect not only interference during replication but also during entry. It also is more complex with regard to both viruses, as they also express their respective structural proteins in a co-infection setting. We were able to demonstrate the restriction of HEV replicons by concurrent HCV replication. Similarly, established HCV replication blocked HEV from replicating, which was revertible upon HCV treatment with DAAs. Of note, the ectopic expression of the HCV NS3/4A protease alone was sufficient to suppress HEV replication, suggesting this as the potential mechanism of action. It is tempting to speculate that NS3/4A might cleave the HEV ORF1 polyprotein, rendering it less functional. We hypothesized that the HEV ORF1 protein might be cleaved by NS3/4A. Because our ORF1 antibodies failed to detect ORF1 in the Western blot, we could not address this question experimentally. However, it is known which part of MAVS [27,42] or TRIF [43], is recognized and, additionally, one study tested a library of small peptides to find 613 sequences that could be cleaved by NS3/4A [44]. Using a bioinformatic approach, we checked if any of these sequences were present in the ORF1 of HEV p6, but none were (data not shown). Hence, cleavage of the HEV polyprotein might likely not be the mechanism of HEV suppression. There have been several epidemiological studies conducted to investigate a link between HCV and HEV infections. Some studies investigated HEV seropositivity in HCV-RNA positive patients with mixed results. A higher HEV seropositivity compared to the control group was reported in one study [45] with another confirming these findings in a mixed cohort of chronic HBV and HCV patients [46], while a third did not find any difference in HEV seropositivity between chronic HCV patients and an aged-matched control group [47]. HCV-IgG was enhanced in HEV-IgG positive persons from the 15,000 US NHANES III study population [48], while an Iranian study of 324 hemodialysis patients did not find a link between HCV and HEV seropositivity [49], as did a study analyzing almost 900 HIV-I infected Spanish patients [50].

To recapitulate HCV/HEV super-infections in vivo, liver humanized mice were utilized. In the HCV super-infection of HEV positive mice, only one mouse was productively infected and vice versa; reduced viral loads of HEV were noted in comparison to the mono-infected group. Two different explanations for this phenomenon seem conceivable. First, that the mutual inhibitory effect of HEV and HCV is due to the first virus eliciting an innate immune response that restricts the latter, irrespective of direct interactions. Second, that the direct interaction is responsible, but it differs between the model systems. If the former were true, then HEV’s innate immune response should be stronger, as HCV is more efficiently restricted by present HEV than vice versa. However, transcriptomic profiling in chimpanzees suggest that it is reverse. Both more genes were differentially expressed and their expression was more robust [51]. Regarding the latter, there are several differences between the model systems. First, we were not able to determine the exact number of infected transplanted PHHs, which was possible in vitro. For HCV patients, it has been described that HCV-infected hepatocytes tend to be found in clusters [52]. Using a laser capture microdissection experimental setup, the fraction of HCV RNA positive cells was reported to range from 21% to 45% in human liver tissue [53], which was similar to that described with antigen staining (7–20%) [52]. The number of HEV-infected hepatocytes in biopsies has been demonstrated to be highly variable, ranging from <2% to 50% in antigen staining and 0–50% in RNA staining [54]. The number of cells positive for each virus in vivo is therefore lower than in cell culture experiments, which might reduce the effect size of inhibitory effects. Secondly, the high variability of hepatocytes positive for either virus could be one reason for the variation of co-infection courses in individual mice. Furthermore, PHHs differ substantially from hepatoma cell lines with respect to innate immunity, host factors and the fact that the former are polarized. For HCV it has been demonstrated that, besides comparatively low antigen levels and replication efficiency [52], the host transcriptional response does differ significantly in chronic HCV patients [55] or PHHs [56] compared to hepatoma cell lines, such as Huh7 [57] or Huh7.5 [58]. Regarding HEV, the infection of PHHs is efficient and transcriptional profiling has been performed recently in PHHs [8], but potential differences between the host response compared to hepatoma cell lines have not been addressed so far. Further studies addressing these differences in HEV–host interplay in different model systems would not only deepen our understanding of HEV pathogenesis, but also of HCV/HEV co-infections, especially in the more complex in vivo situation. In conclusion, co-infection of HCV and HEV was studied for the first time in different experimental model systems, including humanized mice, demonstrating viral interference of HEV and HCV replication. These findings provide new insights into the pathogenesis of HCV/HEV co-infection and may contribute to its clinical management in the future.

## Data Availability

The data presented in this study are available on request from the corresponding author. For the analysis of HCV cleavage sites within the HEV p6 genome, publicly available data were analyzed. This data can be found here: https://journals.plos.org/plosone/article?id=10.1371/journal.pone.0035759#s5, Table S1. Last accessed on 15 January 2022.

## Supplementary Materials

The following supporting information can be downloaded at: https://www.mdpi.com/article/10.3390/cells11060927/s1, Figure S1: HCV/HEV Co-transfection of Huh7.5 cells; Figure S2: VSV super infection of Huh7-Lunet/sg-neo cells; Figure S3: Representative flow cytometry blots and IF pictures of HEV super-transfected Huh7-Lunet; Figure S4: Super-transfection of sofosbuvir-treated Huh7-Lunet cells; Figure S5: Super-transfection of telaprevir-treated Huh7-Lunet cells; Figure S6: HCV super-infections on HEV infected humanized mice.
