# Supplementary material for: Viral Interference of Hepatitis C and E Virus Replication in Novel Experimental Co-Infection Systems
Source: Cells. 2022 Mar 8;11(6):927. doi: 10.3390/cells11060927 (PMC8946046; doi:10.3390/cells11060927)
Supplement: Supplementary file 1 [file cells-11-00927-s001.zip › cells-1576782-supplementary.pdf]

SUPPLEMENTARY MATERIAL TO

“Viral interference of hepatitis C and E virus replication in novel experimental 2 co-infection systems”

SUPPLEMENTARY FIGURES:

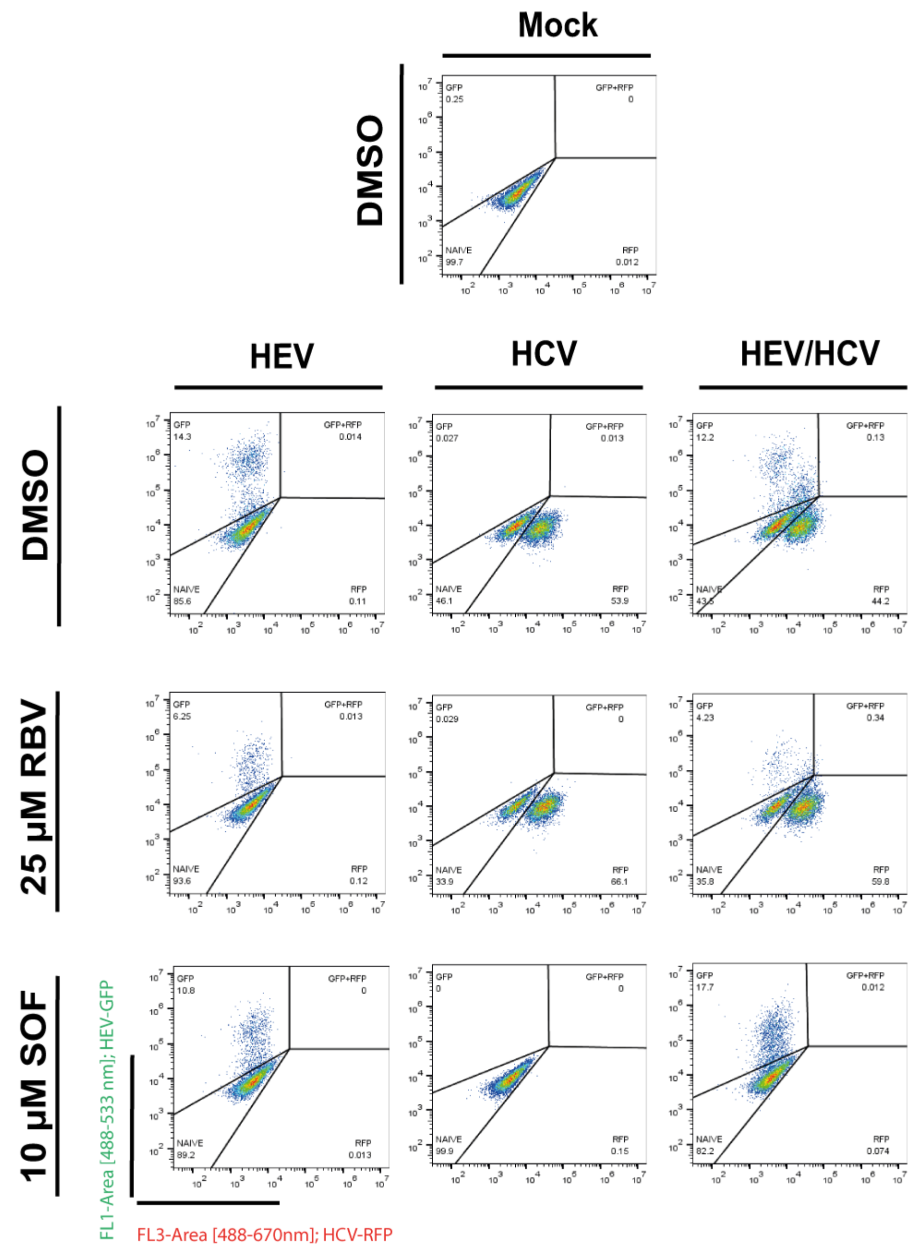

Figure S1. HCV/HEV Co-transfection of Huh7.5 cells. Gating strategy for the flow cytometry analysis to distinguish HEV-positive (upper left corner), HCV-positive (lower right corner) and double positive cells (upper right corner) in double transfected cells in the co-transfection setting. Cells were treated with DMSO, ribavirin (RBV) or sofosbuvir (SOF).

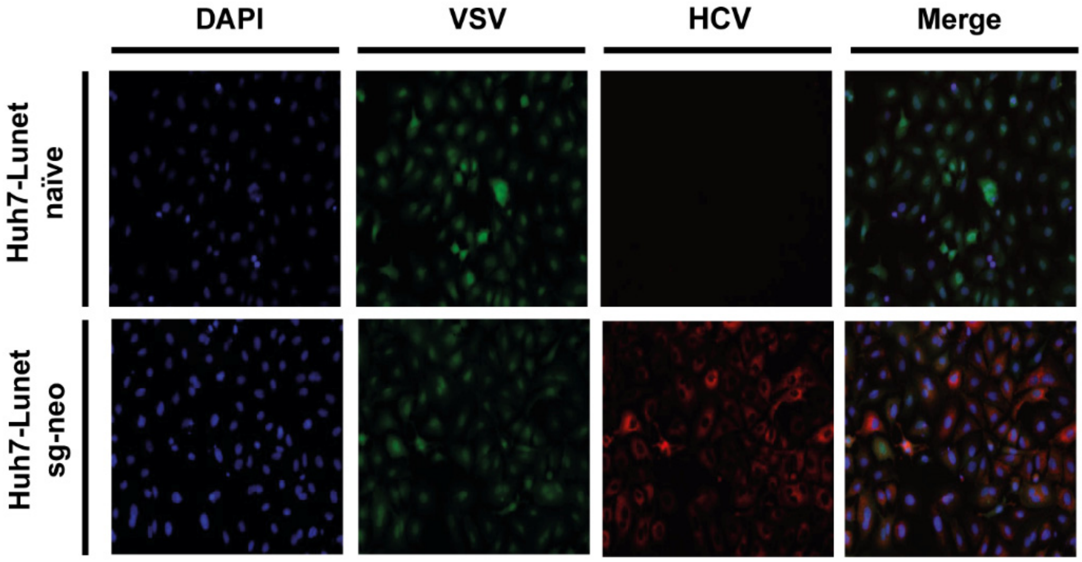

Figure S2. VSV super-infection of Huh7-Lunet/sg-neo cells. Representative IF pictures of Huh7-Lunet naïve or Huh7-Lunet sg/neo infected with VSV.

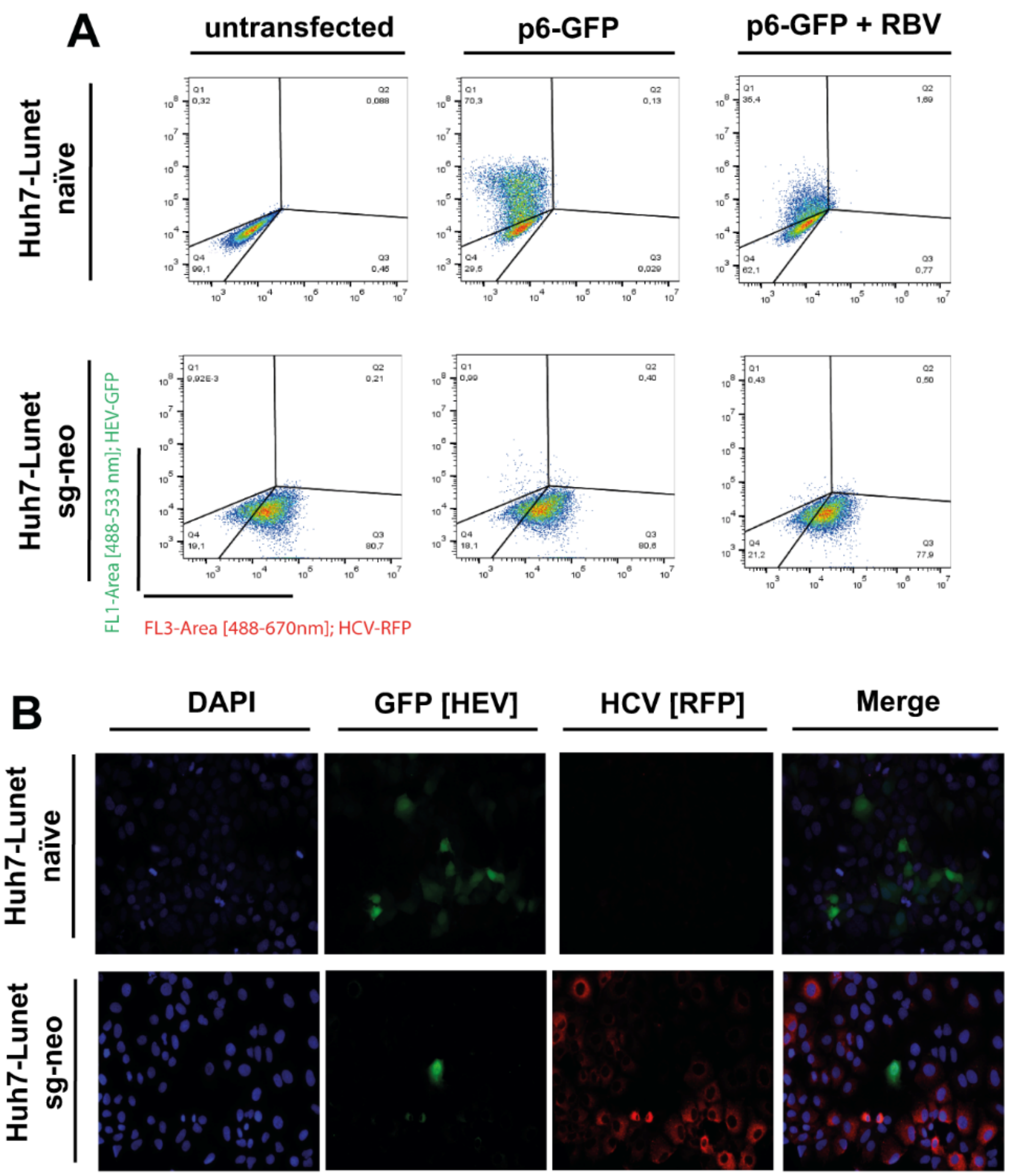

Figure S3. Representative flow cytometry blots and IF pictures of HEV super-transfected Huh7-Lunet. (A) Gating strategy for the flow cytometry analysis to distinguish HEV-positive (upper left corner), HCV-positive (lower right corner) and double positive cells (upper right corner) in super-transfected cells. P6-transfected cells were treated with DMSO or 25 µM ribavirin (RBV). (**B)** Representative IF pictures of HEV transfected Huh7-Lunet naïve cells (upper panels) or HEV super-transfected Huh7-Lunet-sg/neo cells (lower panels).

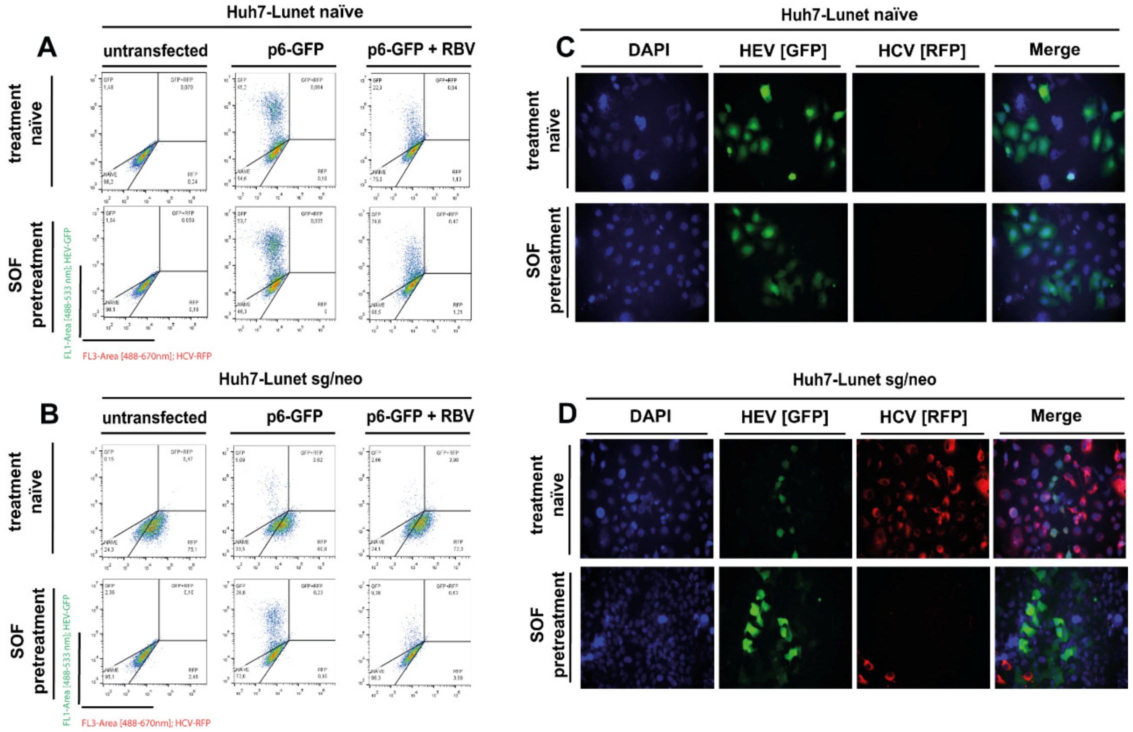

Figure S4. Super-transfection of sofosbuvir-treated Huh7-Lunet cells. (A) Gating strategy for the flow cytometry analysis to distinguish HEV-positive (upper left corner), HCV-positive (lower right corner) and double positive cells (upper right corner) in transfected Huh7-Lunet cells. P6-transfected cells were treated with DMSO or 25 µM ribavirin (RBV) (**B)** Gating strategy for the flow cytometry analysis to distinguish HEV-positive (upper left corner), HCV-positive (lower right corner) and double positive cells (upper right corner) in super-transfected Huh7-Lunet-sg/neo cells. P6-transfected cells were treated with DMSO or 25 µM RBV **C)** Representative IF pictures of HEV transfected Huh7-Lunet naïve cells that have not been pretreated (upper panels) or HEV transfected Huh7-Lunet naïve cells pretreated for 72 h with 10 µM sofosbuvir (SOF) (lower panels). **D)** Representative IF pictures of HEV transfected Huh7-Lunet-sg/neo cells that have not been pretreated (upper panels) or HEV super-transfected Huh7-Lunet-sg/neo cells pretreated with 10 µM SOF (lower panels).

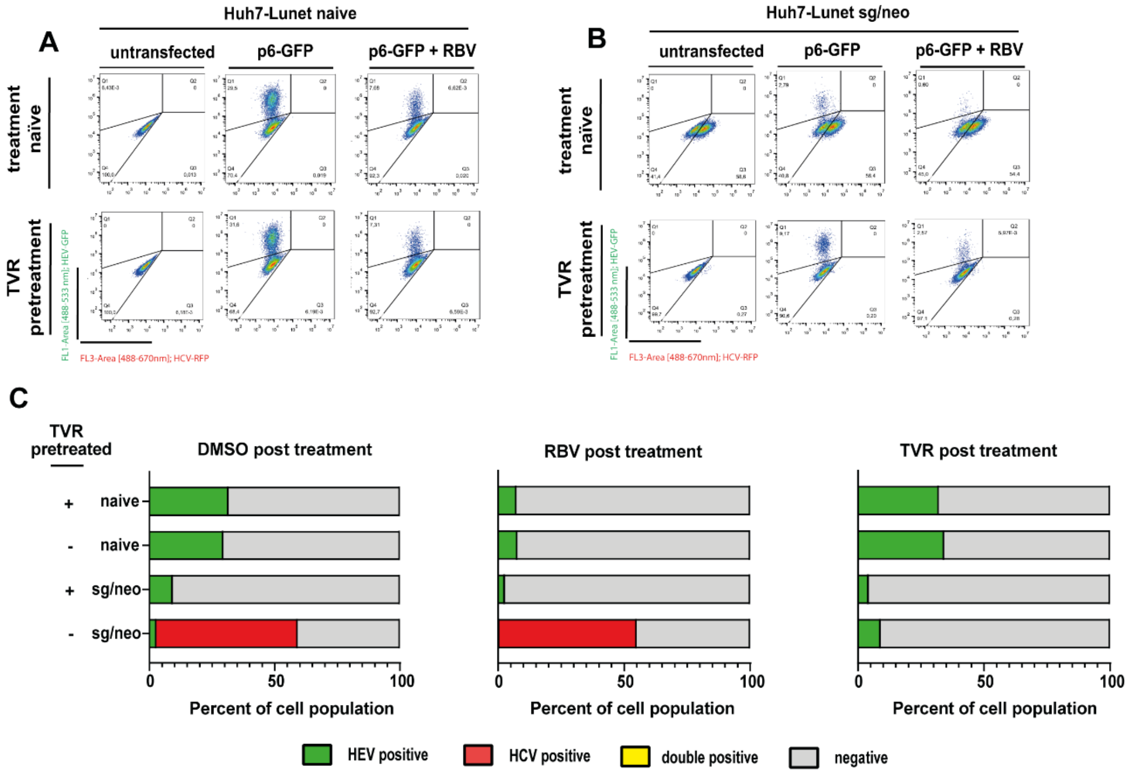

Figure S5. Super-transfection of telaprevir-treated Huh7-Lunet cells (A) Gating strategy for the flow cytometry analysis to distinguish HEV-positive (upper left corner), HCV-positive (lower right corner) and double positive cells (upper right corner) in transfected Huh7-Lunet cells. P6-transfected cells were treated with DMSO or 25 µM ribavirin (RBV) (**B)** Gating strategy for the flow cytometry analysis to distinguish HEV-positive (upper left corner), HCV-positive (lower right corner) and double positive cells (upper right corner) in super-transfected Huh7-Lunet-sg/neo cells. P6-transfected cells were treated with DMSO or 25 µM RBV. (**C)** Huh7-Lunet cells were transfected with JFH1-NS5A-RFP and selected for its stable expression (Huh7-Lunet-sg/neo). Cells were cured by 72 h treatment with 10 µM telaprevir (TVR) or mock treated. Subsequently, cells were electroporated with HEV p6-GFP. Percentage of Huh7-Lunet naïve or Huh7-Lunet/sg-neo cells positive for HCV, HEV, both or none was determined by flow cytometry 5 days post electroporation of HEV subgenomic replicon. Cells were pretreated with DMSO or 10 µM telaprevir (TVR) and subsequently incubated with DMSO, 25 µM RBV or 10 µM TVR.

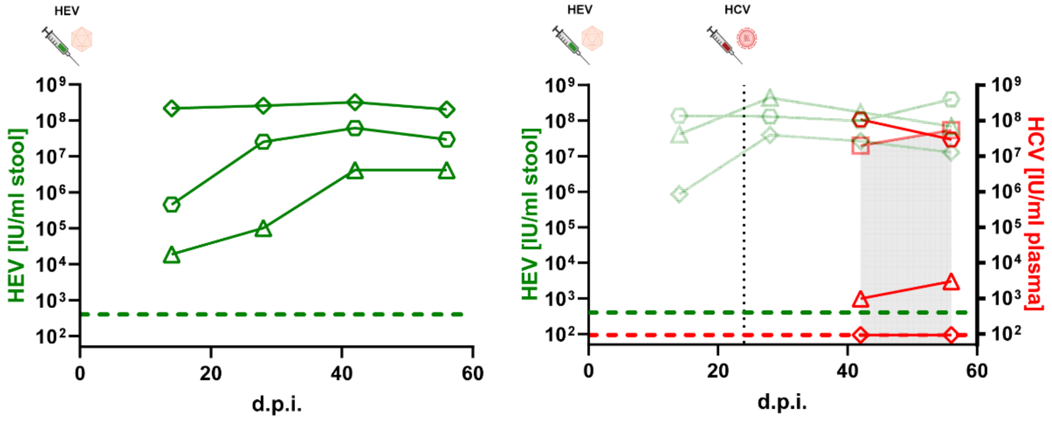

Figure S6. HCV super-infections on HEV-infected humanized mice. Human liver chimeric uPA$^{+/+}$-SCID mice were injected intraperitoneally with HEV and subsequently injected intravenously with HCV (dashed line). HEV RNA (green data points) and HCV RNA (red data points) were periodically measured. Left panel: HEV viral loads of HEV mono-infected mice. Right panel: HEV viral loads of HEV super-infected animals as well as mean HCV titers of HCV mono-infected (semitransparent data points) and HCV titers of HCV super-infected mice. Green dashed line and red dashed line indicate the LOD of the HEV or HCV RT-qPCR, respectively.
